# Supplementary material for: Diversity of carbapenem-resistant Acinetobacter baumannii and bacteriophage-mediated spread of the Oxa23 carbapenemase
Source: Microb Genom. 2022 Feb 1;8(2):000752. doi: 10.1099/mgen.0.000752 (PMC8942029; doi:10.1099/mgen.0.000752)
Supplement: Supplementary material 1 [file mgen-8-0752-s001.pdf]

Figure S1: Maximum-likelihood phylogenetic tree created from portal vertex protein sequences of members of the subfamily Peduovirinae. Sequences were aligned with Clustal Omega and trees constructed using IQTree v1.6.12 with the LG + G4 substitution model IQTree v1.6.12 with ModelFinder, SH-aLRT test and ultrafast bootstrap (1000 replicates). Enterobacteria phage mEp237 (JQ182730) was used as an outgroup to root the tree. Branch length is proportional to the number of substitutions per site (see scale bar). Members of virus genera are denoted by coloured blocks. An asterisk (*) adjacent to a genus name indicate proposed genera that at the time of writing were yet to be ratified by the International Committee on the Taxonomy of Viruses (100).

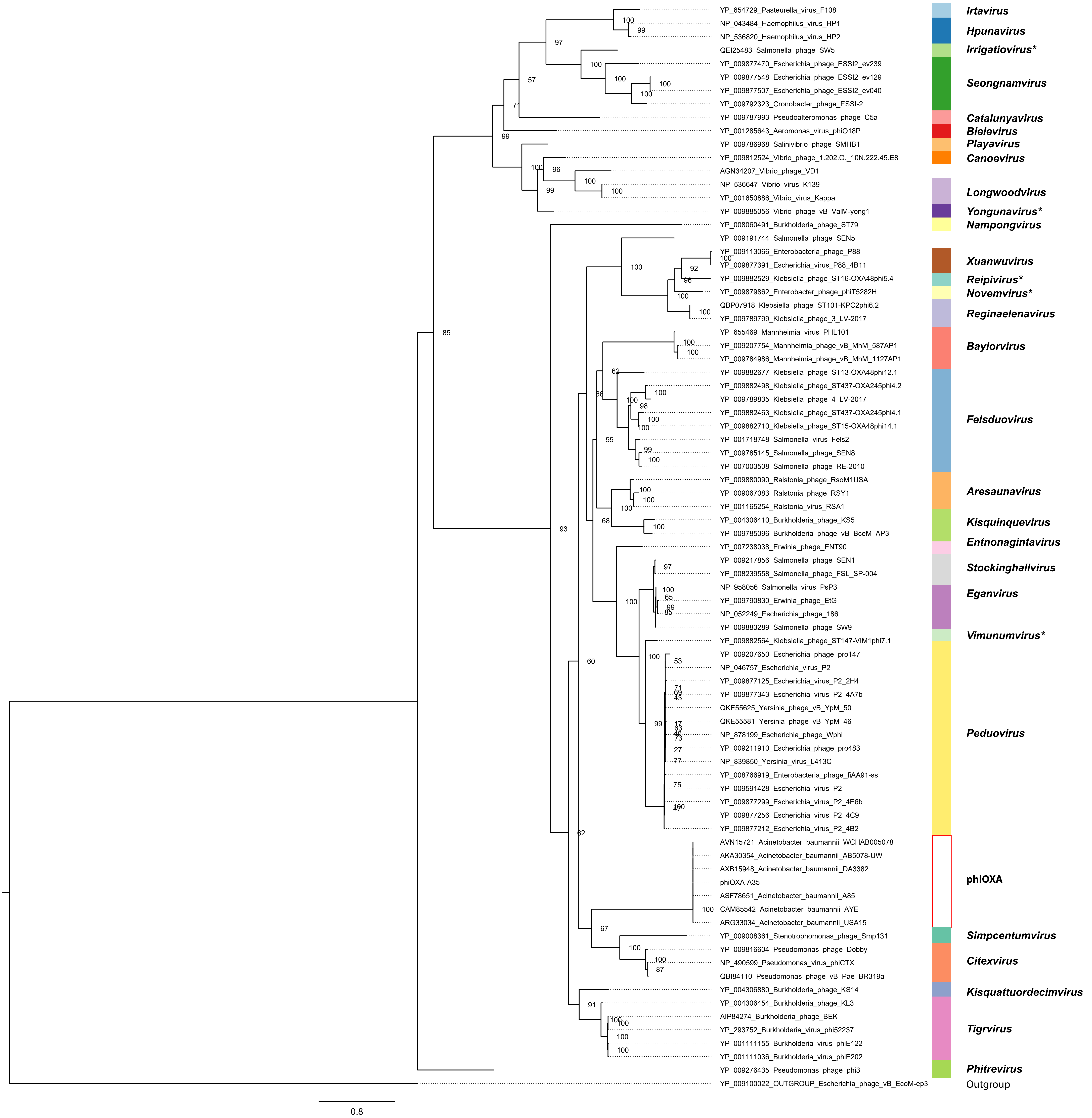
YP_654729_Pasteurella_virus_F108
NP_043484_Haemophilus_virus_HP1
NP_536820_Haemophilus_virus_HP2
QEI25483_Salmonella_phage_SW5
YP_009877470_Escherichia_phage_ESSI2_ev239
YP_009877548_Escherichia_phage_ESSI2_ev129
YP_009877507_Escherichia_phage_ESSI2_ev040
YP_009792323_Cronobacter_phage_ESSI-2
YP_009787993_Pseudoalteromonas_phage_C5a
YP_001285643_Aeromonas_virus_phiO18P
YP_009786968_Salinivibrio_phage_SMHB1
YP_009812524_Vibrio_phage_1.202.O._10N.222.45.E8
AGN34207_Vibrio_phage_VD1
NP_536647_Vibrio_virus_K139
YP_001650886_Vibrio_virus_Kappa
YP_009885056_Vibrio_phage_vB_ValM-yong1
YP_008060491_Burkholderia_phage_ST79
YP_009191744_Salmonella_phage_SEN5
YP_009113066_Enterobacteria_phage_P88
YP_009877391_Escherichia_virus_P88_4B11
YP_009882529_Klebsiella_phage_ST16-OXA48phi5.4
YP_009879862_Enterobacter_phage_phiT5282H
QBP07918_Klebsiella_phage_ST101-KPC2phi6.2
YP_009789799_Klebsiella_phage_3_LV-2017
YP_655469_Mannheimia_virus_PHL101
YP_009207754_Mannheimia_phage_vB_MhM_587AP1
YP_009784986_Mannheimia_phage_vB_MhM_1127AP1
YP_009882677_Klebsiella_phage_ST13-OXA48phi12.1
YP_009882498_Klebsiella_phage_ST437-OXA245phi4.2
YP_009789835_Klebsiella_phage_4_LV-2017
YP_009882463_Klebsiella_phage_ST437-OXA245phi4.1
YP_009882710_Klebsiella_phage_ST15-OXA48phi14.1
YP_001718748_Salmonella_virus_Fels2
YP_009785145_Salmonella_phage_SEN8
YP_007003508_Salmonella_phage_RE-2010
YP_009880090_Ralstonia_phage_RsoM1USA
YP_009067083_Ralstonia_phage_RSY1
YP_001165254_Ralstonia_virus_RSA1
YP_004306410_Burkholderia_phage_KS5
YP_009785096_Burkholderia_phage_vB_BceM_AP3
YP_007238038_Erwinia_phage_ENT90
YP_009217856_Salmonella_phage_SEN1
YP_008239558_Salmonella_phage_FSL_SP-004
NP_958056_Salmonella_virus_PsP3
YP_009790830_Erwinia_phage_EtG
NP_052249_Escherichia_phage_186
YP_009883289_Salmonella_phage_SW9
YP_009882564_Klebsiella_phage_ST147-VIM1phi7.1
YP_009207650_Escherichia_phage_pro147
NP_046757_Escherichia_virus_P2
YP_009877125_Escherichia_virus_P2_2H4
YP_009877343_Escherichia_virus_P2_4A7b
QKE55625_Yersinia_phage_vB_YpM_50
QKE55581_Yersinia_phage_vB_YpM_46
NP_878199_Escherichia_phage_Wphi
YP_009211910_Escherichia_phage_pro483
NP_839850_Yersinia_virus_L413C
YP_008766919_Enterobacteria_phage_fiAA91-ss
YP_009591428_Escherichia_virus_P2
YP_009877299_Escherichia_virus_P2_4E6b
YP_009877256_Escherichia_virus_P2_4C9
YP_009877212_Escherichia_virus_P2_4B2
AVN15721_Acinetobacter_baumannii_WCHAB005078
AKA30354_Acinetobacter_baumannii_AB5078-UW
AXB15948_Acinetobacter_baumannii_DA3382
phiOXA-A35
ASF78651_Acinetobacter_baumannii_A85
CAM85542_Acinetobacter_baumannii_AYE
ARG33034_Acinetobacter_baumannii_USA15
YP_009008361_Stenotrophomonas_phage_Smp131
YP_009816604_Pseudomonas_phage_Dobby
NP_490599_Pseudomonas_virus_phiCTX
QBI84110_Pseudomonas_phage_vB_Pae_BR319a
YP_004306880_Burkholderia_phage_KS14
YP_004306454_Burkholderia_phage_KL3
AIP84274_Burkholderia_phage_BEK
YP_293752_Burkholderia_virus_phi52237
YP_001111155_Burkholderia_virus_phiE122
YP_001111036_Burkholderia_virus_phiE202
YP_009276435_Pseudomonas_phage_phi3
YP_009100022_OUTGROUP_Escherichia_phage_vB_EcoM-ep3
Irtavirus
Hpunavirus
Irrigatiovirus*
Seongnamvirus
Catalunyavirus
Bielevirus
Playavirus
Canoevirus
Longwoodvirus
Yongunavirus*
Nampongvirus
Xuanwuvirus
Reipivirus*
Novemvirus*
Reginaelenavirus
Baylorvirus
Felsduovirus
Aresaunavirus
Kisquinquevirus
Entnonagintavirus
Stockinghallvirus
Eganvirus
Vimunumvirus*
Peduovirus
phiOXA
Simpcentumvirus
Citexvirus
Kisquattuordecimvirus
Tigrvirus
Phitrevirus
Outgroup
0.8

Figure S2: Maximum-likelihood phylogenetic tree created from major capsid protein sequences of members of the subfamily Peduovirinae. Sequences were aligned with Clustal Omega and trees constructed using IQTree v1.6.12 with the WAG + G4 substitution model, SH-aLRT test and ultrafast bootstrap (1000 replicates). Enterobacteria phage mEp237 (JQ182730) was used as an outgroup to root the tree. Branch length is proportional to the number of substitutions per site (see scale bar). Members of virus genera are denoted by coloured blocks. An asterisk (*) adjacent to a genus name indicate proposed genera that at the time of writing were yet to be ratified by the International Committee on the Taxonomy of Viruses (100).

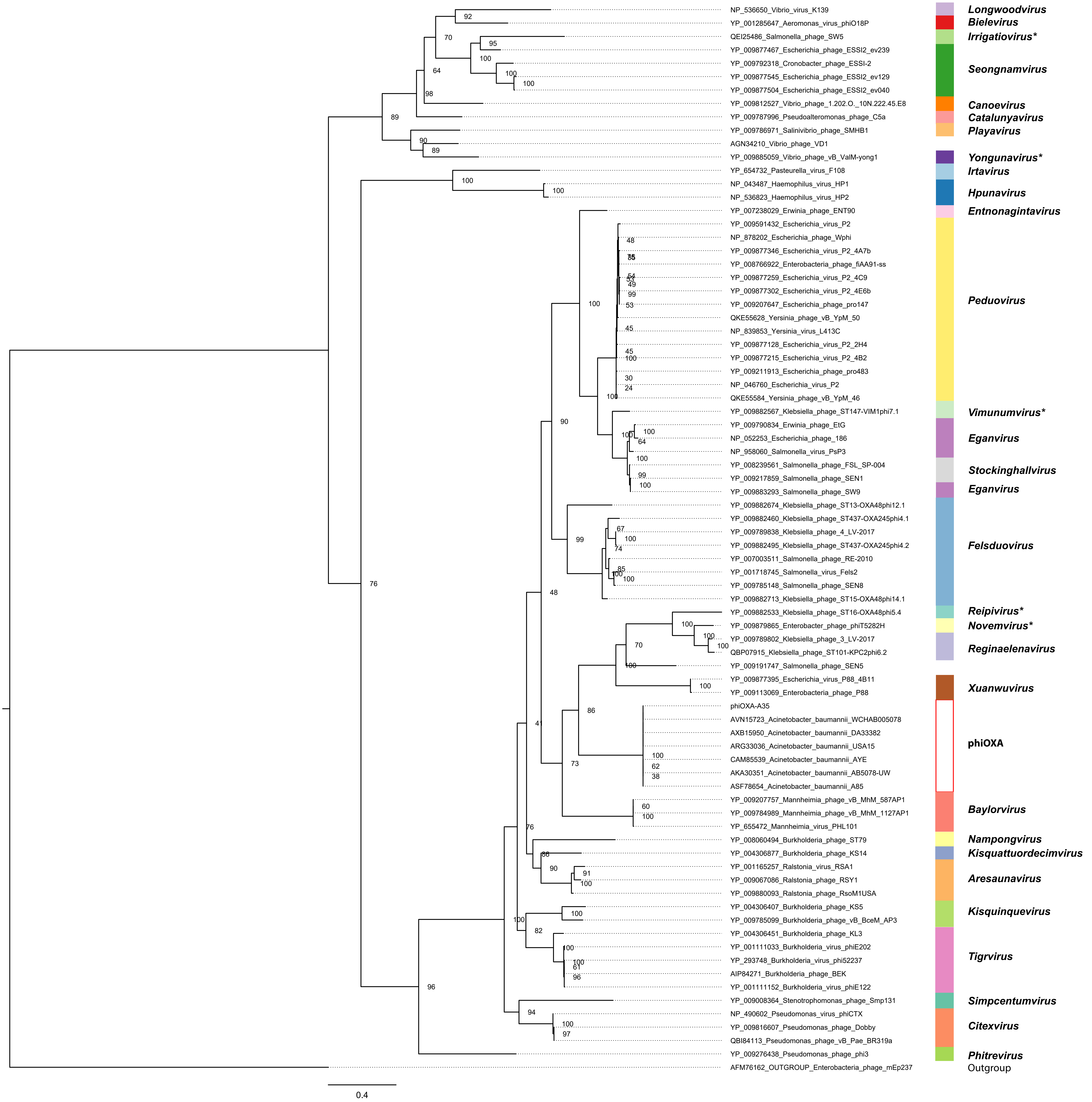
NP_536650_Vibrio_virus_K139
YP_001285647_Aeromonas_virus_phiO18P
QEI25486_Salmonella_phage_SW5
YP_009877467_Escherichia_phage_ESSI2_ev239
YP_009792318_Cronobacter_phage_ESSI-2
YP_009877545_Escherichia_phage_ESSI2_ev129
YP_009877504_Escherichia_phage_ESSI2_ev040
YP_009812527_Vibrio_phage_1.202.O._10N.222.45.E8
YP_009787996_Pseudoalteromonas_phage_C5a
YP_009786971_Salinivibrio_phage_SMHB1
AGN34210_Vibrio_phage_VD1
YP_009885059_Vibrio_phage_vB_ValM-yong1
YP_654732_Pasteurella_virus_F108
NP_043487_Haemophilus_virus_HP1
NP_536823_Haemophilus_virus_HP2
YP_007238029_Erwinia_phage_ENT90
YP_009591432_Escherichia_virus_P2
NP_878202_Escherichia_phage_Wphi
YP_009877346_Escherichia_virus_P2_4A7b
YP_008766922_Enterobacteria_phage_fiAA91-ss
YP_009877259_Escherichia_virus_P2_4C9
YP_009877302_Escherichia_virus_P2_4E6b
YP_009207647_Escherichia_phage_pro147
QKE55628_Yersinia_phage_vB_YpM_50
NP_839853_Yersinia_virus_L413C
YP_009877128_Escherichia_virus_P2_2H4
YP_009877215_Escherichia_virus_P2_4B2
YP_009211913_Escherichia_phage_pro483
NP_046760_Escherichia_virus_P2
QKE55584_Yersinia_phage_vB_YpM_46
YP_009882567_Klebsiella_phage_ST147-VIM1phi7.1
YP_009790834_Erwinia_phage_EtG
NP_052253_Escherichia_phage_186
NP_958060_Salmonella_virus_PsP3
YP_008239561_Salmonella_phage_FSL_SP-004
YP_009217859_Salmonella_phage_SEN1
YP_009883293_Salmonella_phage_SW9
YP_009882674_Klebsiella_phage_ST13-OXA48phi12.1
YP_009882460_Klebsiella_phage_ST437-OXA245phi4.1
YP_009789838_Klebsiella_phage_4_LV-2017
YP_009882495_Klebsiella_phage_ST437-OXA245phi4.2
YP_007003511_Salmonella_phage_RE-2010
YP_001718745_Salmonella_virus_Fels2
YP_009785148_Salmonella_phage_SEN8
YP_009882713_Klebsiella_phage_ST15-OXA48phi14.1
YP_009882533_Klebsiella_phage_ST16-OXA48phi5.4
YP_009879865_Enterobacter_phage_phiT5282H
YP_009789802_Klebsiella_phage_3_LV-2017
QBP07915_Klebsiella_phage_ST101-KPC2phi6.2
YP_009191747_Salmonella_phage_SEN5
YP_009877395_Escherichia_virus_P88_4B11
YP_009113069_Enterobacteria_phage_P88
phiOXA-A35
AVN15723_Acinetobacter_baumannii_WCHAB005078
AXB15950_Acinetobacter_baumannii_DA33382
ARG33036_Acinetobacter_baumannii_USA15
CAM85539_Acinetobacter_baumannii_AYE
AKA30351_Acinetobacter_baumannii_AB5078-UW
ASF78654_Acinetobacter_baumannii_A85
YP_009207757_Mannheimia_phage_vB_MhM_587AP1
YP_009784989_Mannheimia_phage_vB_MhM_1127AP1
YP_655472_Mannheimia_virus_PHL101
YP_008060494_Burkholderia_phage_ST79
YP_004306877_Burkholderia_phage_KS14
YP_001165257_Ralstonia_virus_RSA1
YP_009067086_Ralstonia_phage_RSY1
YP_009880093_Ralstonia_phage_RsoM1USA
YP_004306407_Burkholderia_phage_KS5
YP_009785099_Burkholderia_phage_vB_BceM_AP3
YP_004306451_Burkholderia_phage_KL3
YP_001111033_Burkholderia_virus_phiE202
YP_293748_Burkholderia_virus_phi52237
AIP84271_Burkholderia_phage_BEK
YP_001111152_Burkholderia_virus_phiE122
YP_009008364_Stenotrophomonas_phage_Smp131
NP_490602_Pseudomonas_virus_phiCTX
YP_009816607_Pseudomonas_phage_Dobby
QBI84113_Pseudomonas_phage_vB_Pae_BR319a
YP_009276438_Pseudomonas_phage_phi3
AFM76162_OUTGROUP_Enterobacteria_phage_mEp237
Longwoodvirus
Bielevirus
Irrigatiovirus*
Seongnamvirus
Canoevirus
Catalunyavirus
Playavirus
Yongunavirus*
Irtavirus
Hpunavirus
Entnonagintavirus
Peduovirus
Vimunumvirus*
Eganvirus
Stockinghallvirus
Eganvirus
Felsduovirus
Reipivirus*
Novemvirus*
Reginaelenavirus
Xuanwuvirus
phiOXA
Baylorvirus
Nampongvirus
Kisquattuordecimvirus
Aresaunavirus
Kisquinquevirus
Tigrvirus
Simpcentumvirus
Citexvirus
Phitrevirus
Outgroup
0.4

Figure S3: Distribution of different inserted elements at the tRNA-Leu site in $CC^{PAS}1$ isolates. The tree is the same as that shown in Figure 1, retaining only those leaves representing $CC^{PAS}1$ isolates. Leaves are annotated with the type of insertion found at tRNA-Leu: orange indicates phiOXA, pale blue indicates no insertion, and pale pink indicates an insertion other than phiOXA is present. In the case of strain AYE the insertion is bacteriophage RPHR (2) which is identical to phiOXA except for the IS*Aba1-oxa23* insertion (see Figure 4), and in isolates 6322619 and 6322626 this represents the insertion of an unrelated uncharacterised element.

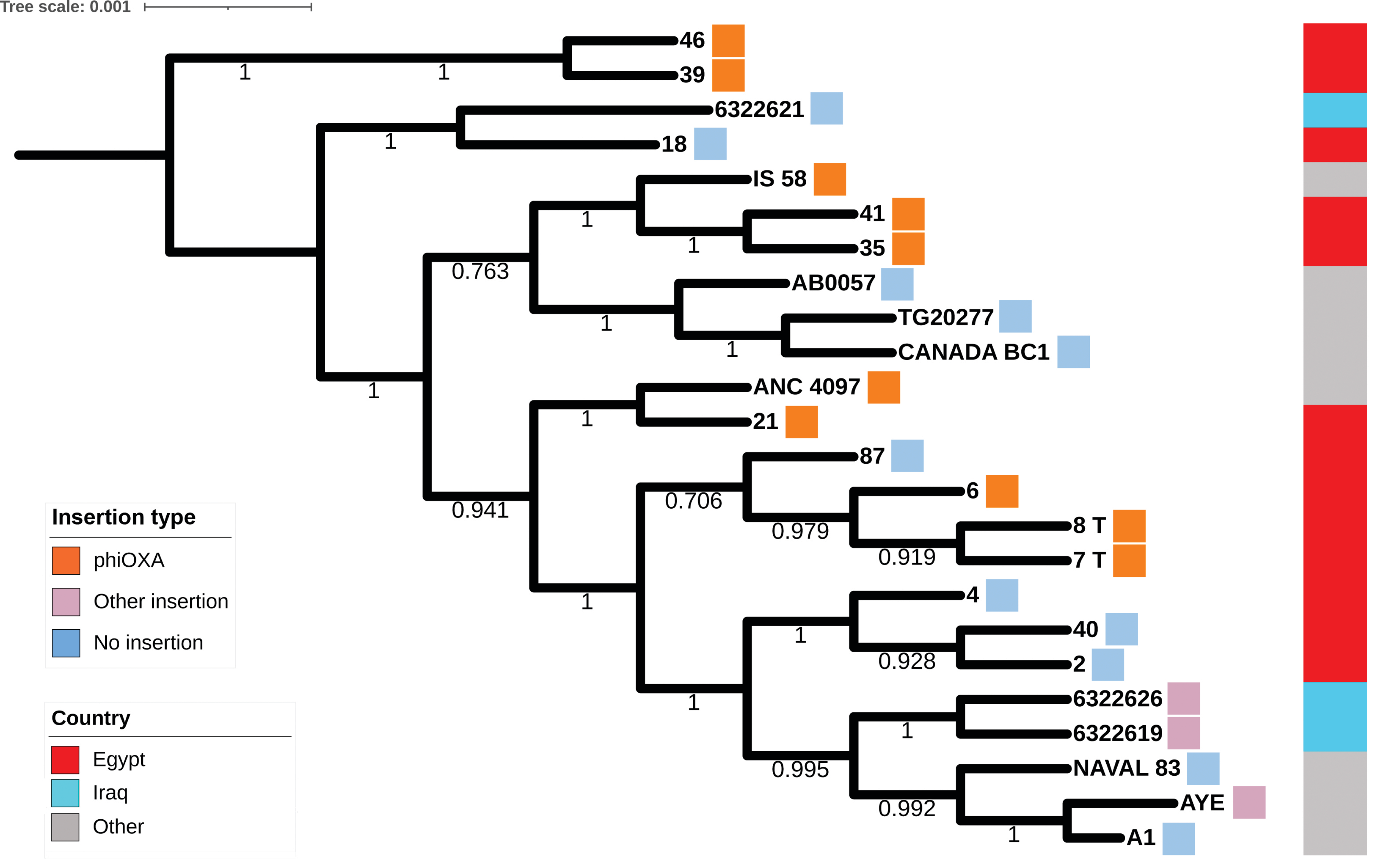

Tree scale: 0.001
46
39
6322621
18
IS 58
41
35
AB0057
TG20277
CANADA BC1
ANC 4097
21
87
6
8 T
7 T
4
40
2
6322626
6322619
NAVAL 83
AYE
A1
0.763
0.941
0.706
0.979
0.919
0.928
0.995
0.992
Insertion type
phiOXA
Other insertion
No insertion
Country
Egypt
Iraq
Other

References

1. Van Zyl L, Lueder M, Bishop-Lilly K, Turner D, Adriaenssens E, et al. Create five new genera and 29 species in the subfamily *Peduovirinae* 2019.002D; 2019. https://ictv.global/ICTV/proposals/2020

2. Vallenet D, Nordmann P, Barbe V, Poirel L, Mangenot S, et al. Comparative analysis of *Acinetobacters*: three genomes for three lifestyles. *PLoS One* 2008;3:e1805. 10.1371/journal.pone.0001805
